# Therapeutic Effect of Acetabular Fractures Using the Pararectus Approach Combined with 3D Printing Technique

**DOI:** 10.1111/os.12738

**Published:** 2020-10-28

**Authors:** Ruyi Zou, Min Wu, Jianzhong Guan, Yuzhou Xiao, Xiaotian Chen

**Affiliations:** ^1^ Department of Orthopaedics The First Affiliated Hospital of Bengbu Medical College Bengbu China

**Keywords:** 3D printing, Acetabular fractures, Fracture fixation, Pararectus approach

## Abstract

**Objective:**

To explore the clinical efficacy of pararectus approach combined with 3D printing technique for the surgical treatment of partial acetabular fractures.

**Methods:**

We retrospectively evaluated 33 (20 males and 13 females) patients with acetabular fractures in the period of June 2017 to December 2018. According to Judet and Letournel classification: 11 cases were of anterior column fracture, 10 cases were of double column fracture, seven fractures were of the anterior column with posterior half transverse, three fractures were of transverse fracture, and two cases were of “T” fracture. For all cases, 3D printing is used to print the acetabular model. Pre‐bent reconstruction plates from the model were placed to fixate fractures via the pararectus approach.

**Results:**

Thirty‐three patients (mean age 48 years; range, 35–63 years), included 20 men and 13 women, were treated successfully with open reduction and internal fixation by the pararectus approach. Surgery duration was 203 min on average (range: 135–245 min), and intra‐operative bleeding was 1030 mL on average (range: 450–1400 mL). All patients were followed‐up for 12–18 months (average,14 months); two patients (6.0%) developed postoperative ossifying myositis, and there are no obvious symptoms at present; one patient (3.0%) developed postoperative wound infection, and the wound completely improved by secretion culture, enhanced dressing, and effective antibiotics; all the acetabular fractures united after 12 to 16 weeks (average,13 weeks). According to the modified Merle d'Aubigne and Postel scoring system to assess the hip function: excellent in 22 cases (66.7%), good in seven cases (21.2%), and fair in four cases (12.1%).

**Conclusions:**

In the treatment of partial acetabular fractures, the pararectus approach combined with 3D printing technique can achieve effective reduction and fixation, decrease intraoperative hemorrhage, shorten operation time, and the internal fixation position can be properly adjusted during the operation by looking directly at the model.

## Introduction

Acetabular fractures are usually associated with some high‐energy injuries. Due to its exceptional location, open reduction and internal fixation has become the “gold standard” for the treatment of displaced acetabular fractures^1^, ^2^, ^3^. The position of the acetabulum is deep, and it is often difficult to expose fractures, especially complex acetabular fractures.

In the classic work of Judet and Letournel, the ilioinguinal approach was described in detail. Owing to this surgical approach, surgeons can fully expose the front of the acetabulum, meaning it has always been a classic approach to the treatment of acetabular anterior fractures^4^, ^5^, ^6^, ^7^. However, this surgical approach has a long incision and often needs to dissect important nerves, blood vessels, and other structures, so the operation is complicated. Therefore, some scholars reported that the improved Stoppa was used as a small incision to replace the traditional ilioinguinal approach^8^, ^9^, ^10^, ^11^. A recent study showed that compared with the ilioinguinal approach, reduction and fixation of fractures can be achieved more easily by using the modified Stoppa approach^12^. But, the Stoppa way still has certain limitations. For example, there is a specified distance from the iliac wing fracture. It is often to use a tiny incision on the iliac crest to assist in the reduction. At the same time, because of the small incision, it is difficult to operate in patients with severe displacement of the fracture, especially in obese patients.

To overcome the limitations of these conventional approaches, we used the pararectus approach described by Keel^13^. At the same time, in recent years, 3D printing technology has been increasingly utilized in cases of complex fractures. So, we use the pararectus approach in combination with 3D printing technology to treat acetabular fractures. We hope that this approach can: fully reveal the acetabular fracture; directly look at important structures such as blood vessels and nerves during operation; and cause less soft tissue damage. According to the preoperative 3D printing model, the fracture morphology of the acetabulum can be visually observed and the steel plates can be pre‐bent before surgery.

## Materials and Methods

This study was approved by the local ethics committee of our institution, and all patients gave informed consent and signed it. We found patients through the institution's case system and collected data through outpatient review.

**Fig. 1:**
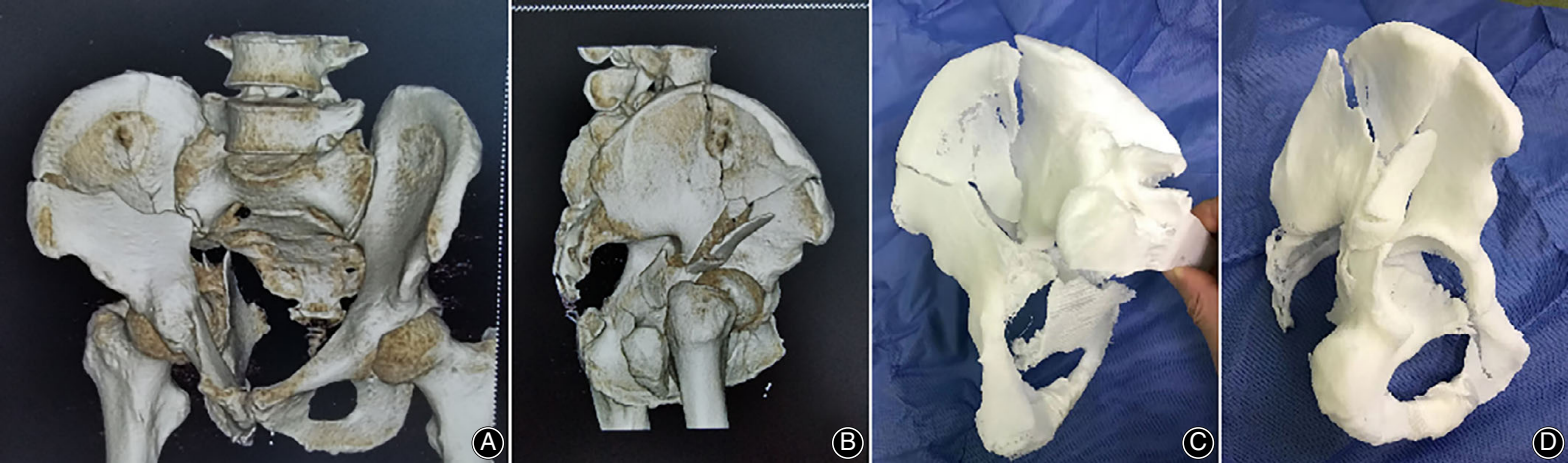
(A, B) A 45‐year‐old male patient suffered a road‐traffic accident. The fracture was diagnosed as a double column fracture through took CT scan +3D reconstruction. (C, D) 3D printed model made from data obtained as a result of CT scan.

Patients who fulfilled the following inclusion criteria were included in the study: (i) admissions were completed with pelvic film, pelvic computed tomography (CT) scan and 3D reconstruction and 3D printing model; (ii) closed acetabular fracture; (iii) regular follow‐up after discharge. Exclusion criteria included: (i) open acetabular fracture; and (ii) those with deformity and dysfunction of lower limbs before injury. We proceeded to a retrospective analysis of 33 patients treated at our institution from June 2017 to December 2018. According to the Judet and Letournel classification: 11 cases were of anterior column fracture, 10 cases were of double column fracture, seven fractures were of the anterior column with posterior half transverse, three fractures were of transverse fracture, and two case were of “T” fracture. All patients were operated on by the corresponding author (Min Wu). All patients underwent a pelvic CT scan to obtain the DICOM format data of the 3D reconstructed image of the pelvic thin layer, imported into Mimics10.01 software (Materialise company, Belgium) to generate a 3D virtual model of the pelvis; then using fused deposition modeling 1: 1 scale printing to create a mirrored pelvic model (Fig. 1). Patient demographics are depicted in Table 1.

**TABLE 1:** Patient demographics

Parameter	Value	Percent (%)
Mean age(years)	48 (35–63)	
Gender		
Male	20	60.6
Female	13	39.4
Mechanism of injury		
Road traffic accident	18	54.6
Fall	11	33.3
Heavy object injury	4	12.1
Fracture classifification		
Anterior column	11	33.3
Both columns	10	30.3
Anterior column and posterior hemitransverse	7	21.2
Transverse	3	9.1
T‐Type	2	6.1
Mean delay to surgery (days)	9 (6–17)	

### *Surgical Technique*

**Fig. 2:**
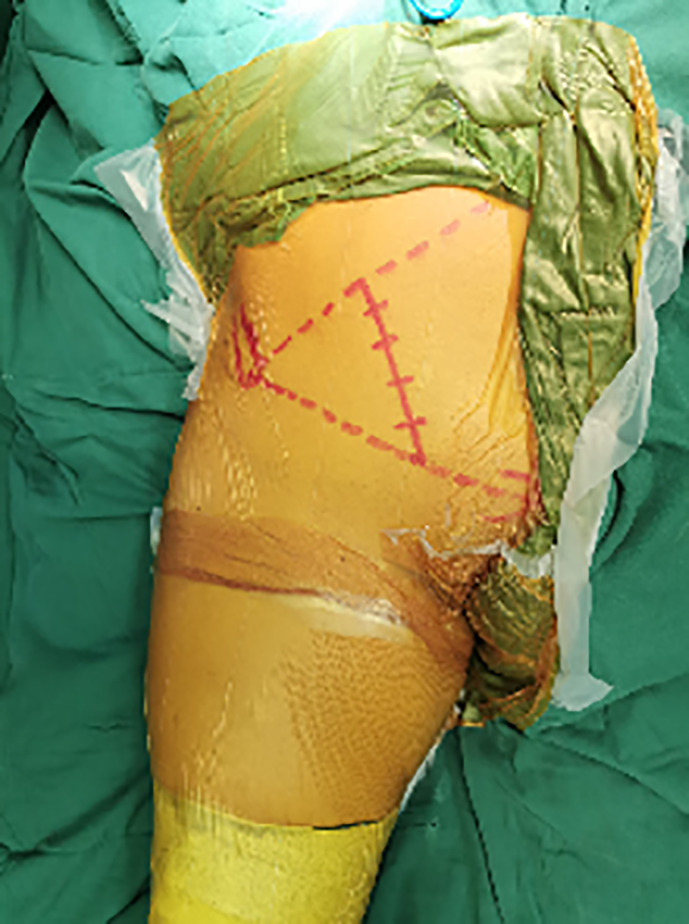
Landmarks and the skin incision of the pararectus approach. The incision start at the middle and outer 1/3 of the line connecting the umbilical cord with the ASIS, and stop at the middle and inner 1/3 of the line connecting the ASIS with pubic symphysis.

**Fig. 3:**
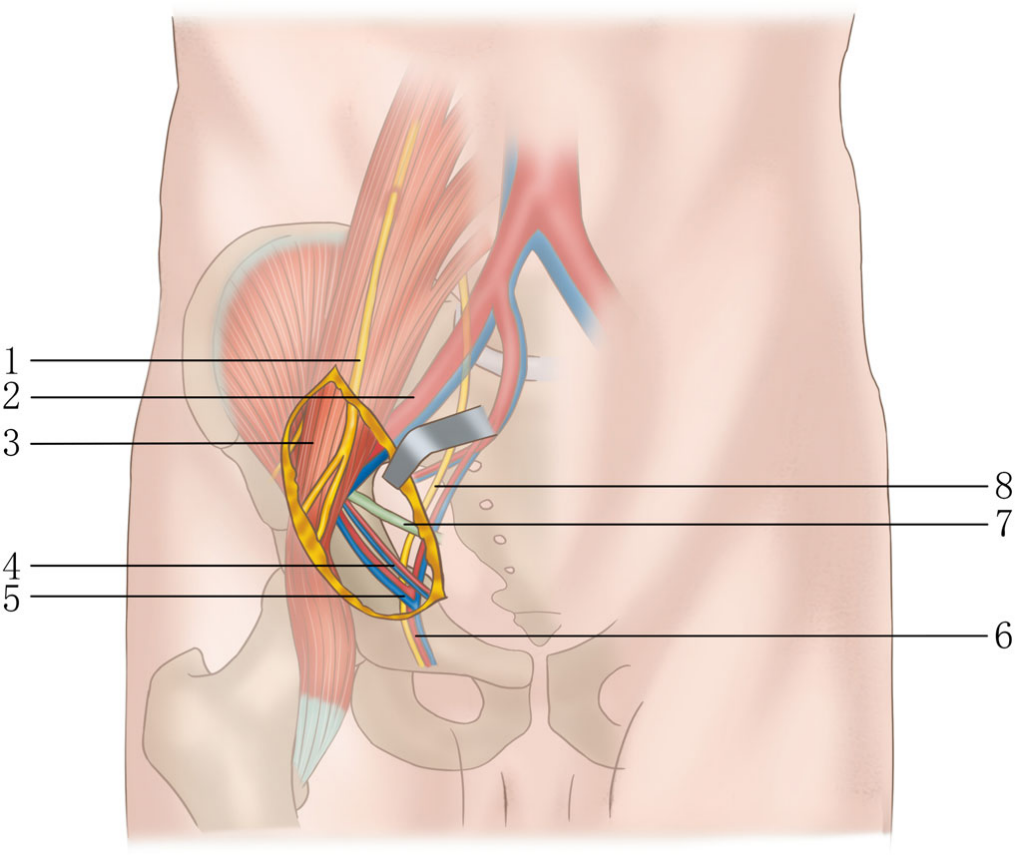
Schematic drawing showing surgical exposure using the Pararectus approach. Through the schematic diagram, we can clearly see the scope of the operation area and the important neurovascular. 1,genital femoral nerve; 2,external iliac artery/vein; 3,iliac psoas muscle; 4,inferior abdominal artery/vein; 5,”death crown”;6,obturator vessels; 7,spermatic cord in men or round ligament in women; 8,obturator nerve.

Landmarks for the incision were the navel, pubic symphysis and the anterior superior iliac spine (ASIS), as professor Keel^13^ described. After general anesthesia, the incision starts at the middle and outer 1/3 of the line connecting the umbilical cord with the ASIS, and stops at the middle and inner 1/3 of the line connecting the ASIS with pubic symphysis (Fig. 2). The length of this incision was about 9–14 cm. Along the incision line, the skin, subcutaneous tissue and deep fascia were cut in turn. Palpate to identify the outer edge of rectus abdominous and cut the external oblique, internal oblique and transverse abdominal muscles along it. We can clearly see important neurovascular structures (Fig. 3). Pay heed to avoid damaging the blood vessels and spermatic cord (or round ligament of the uterus). Through the retroperitoneal space, the peritoneum and the pelvic tissue are led to the inside. Other structures are led to the outside, and the real pelvic ring structure is exposed to the inside of the pelvis. Searching for the “death crown” above the obturator of the superior pubic branch and ligate it.

According to the need for reduction of fractures, different windows are selected for exposure. The acetabular fractures reduction was completed with apical cone, reduction forceps and Kirschner wire during the operation. The Kirschner wire was used to temporarily fix the fracture block. According to the 3D printing model, the reconstruction plates and screw were selected at appropriate positions. When satisfied, leave the drainage tube and suture layer by layer.

### *Evaluation*

**Fig. 4:**
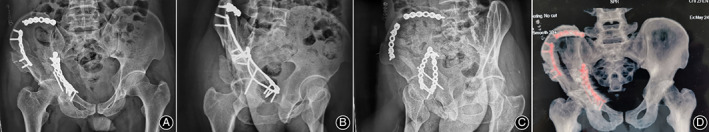
Postoperative anteroposterior (AP) (A) and Judet oblique view x‐rays (B to C), and computed tomography (CT) scan (D) of the acetabulum shows the outcome of reduction of the fracture is well.

All surgical data (blood loss, operative time, length of the incision, intraoperative and postoperative complications) were recorded. Patients were regularly followed up at one, three, six, and 12 months. All patients underwent anterior posterior (AP) and Judet oblique view x‐rays projections of acetabular fractures, and pelvic CT+ three‐dimensional reconstruction after surgery (Fig. 4), corresponding author (Wu Min) evaluated the x‐rays of all patients, suggesting that fracture reduction is well. Clinical outcomes were evaluated using the modified Merle d'Aubigné score, the modified Merle d'Aubigné score system mainly includes three aspects as pain, movement, and walking, ranging from excellent (18 points), good (15–17 points), fair (14 or 13 points), and poor (<13 points).

## Results

Thirty‐three patients (mean age 48 years; range, 35–63 years), included 20 men and 13 women, were treated successfully with open reduction and internal fixation by the pararectus approach. The average time of surgery was 203 min (range: 135–245 min) and mean intraoperative blood loss was 1030 mL (range: 450‐1400 mL); 33 patients were followed up for 12–18 months (average,14 months); the average length of the incision was 11 cm (range: 9‐14 cm). Two patients (6.0%) developed postoperative ossifying myositis, and there are no obvious symptoms at present; one patient (3.0%) developed postoperative wound infection, and the wound completely improved by secretion culture, enhanced dressing and effective antibiotics; all patients underwent pelvic film and pelvic CT and 3D reconstruction after surgery, suggesting that fractures reduction as well. All the acetabular fractures united after 12 to 16 weeks (average,13 weeks); according to the modified Merle d'Aubigne and Postel scoring system to assess the hip function: excellent in 22 cases (66.7%), good in seven cases(21.2%), and fair in four cases(12.1%). Specific data refer to Table 2.

**TABLE 2:** Postoperative outcomes

Postoperative data	Value	Percent
Mean blood loss(ml)	1030 (range: 450–1400)	
Mean operative time(min)	203 (range: 135–245)	
Mean length of incision(cm)	11 (range: 9–14)	
Mean union time(weeks)	13 (range: 12–16)	
Postoperative complications		
Postoperative ossifying myositis	2	6.1
Postoperative wound infection	1	3.0
The modified Merle d'Aubigné score		
Excellent	22	66.7
Good	7	21.2
Fair	4	12.1

## Discussion

The pararectus approach combined with 3D printing technique was introduced for treatment of acetabular fractures. The main suggested advantage in comparison to conventional approaches was to simplify the treatment of specific fracture patterns with less invasive tissue dissection. The pararectus approach can be used to fix the acetabular anterior and quadrilateral acetabular fractures under direct vision. Additionally, application of 3D printing technique can directly observe the fracture morphology of the acetabulum and pre‐bend of the steel plates before surgery.

Due to the special anatomical position of the acetabulum and its relationship with surrounding tissues, the anatomical reduction of the articular surface has become the treatment target for displaced acetabular fractures^14^, ^15^, ^16^. Appropriate surgical approach can not only reduce the injury of the patient, shorten the operation time, but also realize the visualization of the fracture, which helps the reduction and fixation of the fracture.

The pararectus approach, as reported by Keel in 2012, was used to treat acetabular fractures predominantly involving the anterior column and the quadrilateral plate^13^. Compared with the traditional ilioinguinal approach and the modified Stoppa approach, the pararectus approach has the advantages of less trauma, no need to dissect important nerve and blood vessels, and less damage to tissues. At the same time, because the incision is located on the same side of the fracture, it is closer to the acetabulum and can look directly at the front of the acetabulum, which is of great significance for the reduction and fixation of the fracture^13^, ^17^.

Keel *et al*. reported that in the treatment of 48 cases of acetabular fractures, the pararectus approach was used to provide clear fracture visualization; the average incision length was 11 cm, which reduced soft tissue damage^7^. Bastian *et al*. showed that, compared with the modified Stoppa approach, the pararectus approach can reveal more false pelvis, and the posterior ring can be fixed without additional surgical approach^18^. Mardian *et al*. reported that in the comparative study of the pararectus approach and the ilioinguinal approach, the pararectus approach was superior to the ilioinguinal approach in reducing the gap between the fracture blocks^17^.

As the application of 3D printing technology in orthopaedics becomes more and more mature, when we understand the classification and displacement of fracture, the process of getting rid of the traditional 2D imaging data obtained only before surgery and instead constructing a 3D morphology in mind. The 3D printed fracture model can be more convenient to observe the shape of the fracture block, which helps orthopaedic surgeons to stereoscopically locate the acetabular fracture. At the same time, the application of 3D printing technology can enable orthopaedic surgeons to pre‐design the fracture reduction sequence, the position of the steel plate and the screwing angle of the screw according to the characteristics of the fracture. In studies by Tack and Martellini *et al*. the application of 3D printing technology reduced the patient's operation time, intraoperative blood loss, and intraoperative and postoperative complications^19^, ^20^.

When dealing with acetabular fractures, we often need a complete preoperative examination, preoperative evaluation, and choice of surgical approach. Attention should be paid to the treatment of acetabulum fracture by the pararectus approach, including: (i) a single incision cannot be used for fractures of the posterior wall with acetabular joints, and a combined Kocher–Langenbeck approach is often required. For patients who need the Kocher–Langenbeck combined approach, adopt a “floating” position before disinfecting the towels, which is conducive to changing to the supine or lateral position as needed during the operation and reducing the operation time; (ii) for patients with severe extra peritoneal adhesions, consider using this surgical approach as appropriate; (iii) the surgeon must be familiar with the anatomy of the abdomen, and in patients with peritoneal rupture, suture it in time^13^, ^21^; “death crown” blood vessels are the anastomotic arteriovenous system of the inferior abdominal wall or the external iliac arteriovenous system and the obturator artery and vein^22^, ^23^. This surgical approach can view it directly above the medial obturator of the superior pubic branch; once found, it should be ligated to prevent the tear of the blood vessel caused by traction during fracture reduction, resulting in uncontrollable bleeding^23^, ^24^.

The main limitation of this study is its small size from a single institution. Owing to the relatively short follow‐up time in this study, the mid‐ to long‐term clinical efficacy requires further follow‐up of patients.
